# CRISPR‐Cereal: a guide RNA design tool integrating regulome and genomic variation for wheat, maize and rice

**DOI:** 10.1111/pbi.13675

**Published:** 2021-08-14

**Authors:** Chao He, Hao Liu, Dijun Chen, Wen‐Zhao Xie, Mengxin Wang, Yuqi Li, Xin Gong, Wenhao Yan, Ling‐Ling Chen

**Affiliations:** ^1^ National Key Laboratory of Crop Genetic Improvement Hubei Hongshan Laboratory Huazhong Agricultural University Wuhan China; ^2^ National Key Laboratory of Crop Genetic Improvement Huazhong Agricultural University Wuhan China; ^3^ School of Life Sciences Nanjing University Nanjing China; ^4^ State Key Laboratory for Conservation and Utilization of Subtropical Agro‐bioresources College of Life Science and Technology Guangxi University Nanning China

**Keywords:** Crop gene editing, gRNA design, regulome, open chromatin, SNP

The clustered regularly interspaced short palindromic repeat (CRISPR)‐associated protein (Cas) genome editing system (CRISPR‐Cas) is revolutionizing agriculture. In this system, a guide sequence that matches to a particular genomic DNA is placed in front of a synthetic RNA that consists of a scaffold sequence necessary for Cas‐binding to form a guide RNA (gRNA). gRNA/Cas complex binds to the target DNA that contains a protospacer adjacent motif (PAM) via base‐paring and generates a double‐strand break (DSB) by Cas protein. Mutations will be created when the DSB cannot be perfectly repaired. Among kinds of Cas variants, Cas9 and Cas12a (also termed Cpf1) are the two major nucleases with highest edit efficiency. NGG (N = A, T, G or C) for SpCas9 from *Streptococcus pyogenes*, TTTN for Cpf1 from *Acidaminococcus* or *Lachnospiraceae,* is necessary for recruiting the nuclease to produce DSBs.

The efficiency of CRISPR‐Cas is largely determined by the sequence of gRNA and the chromatin status of target region. Guide RNA devotes to direct the CRISPR‐Cas to the target editing (on‐target) sites and Cas protein binds to open chromatin with higher affinity thus resulting in higher efficiency. In addition, nucleotide polymorphisms in guide sequence greatly affect editing efficiency. To date, a number of CRISPR gRNA design tools have been developed but hardly include other information than base‐paring. A gRNA design tool that quickly scans the genome for on‐targets and off‐targets, and considers chromatin accessibility and single nucleotide polymorphisms (SNPs) at target region is highly demanded, especially for wheat with a gigantic genome.

To address the above issues, we developed CRISPR‐Cereal, a web‐based gRNA design tool integrates the information of gene expression profile, chromatin status including chromatin openness and histone modifications, and SNP variations of the putative targets for three leading crops, wheat (*Triticum aestivum*), maize (*Zea mays*) and rice (*Orazy sativa*) (Figure 1a,b). The genome of wheat IWGSCv1.1 (Appels *et al*., 2018), *O. sativa* subsp. *indica* cv Minghui63 (MHRS3) (Song *et al*., 2021), *O. sativa* subsp. *japonica* cv Nipponbare (IRGSP‐1.0) and the B73 maize genome AGPv4 (Jiao *et al*., 2017) are used as reference genomes. Notably, the MHRS3 genome is the first gap‐free genome in crops, which allows gRNA design for ‘dark area’ like centromeric region (Figure 1b). The gap‐free genome MHRS3 unmasks more than 395 non‐TE genes located in centromere regions (Song *et al*., 2021).

**Figure 1:**
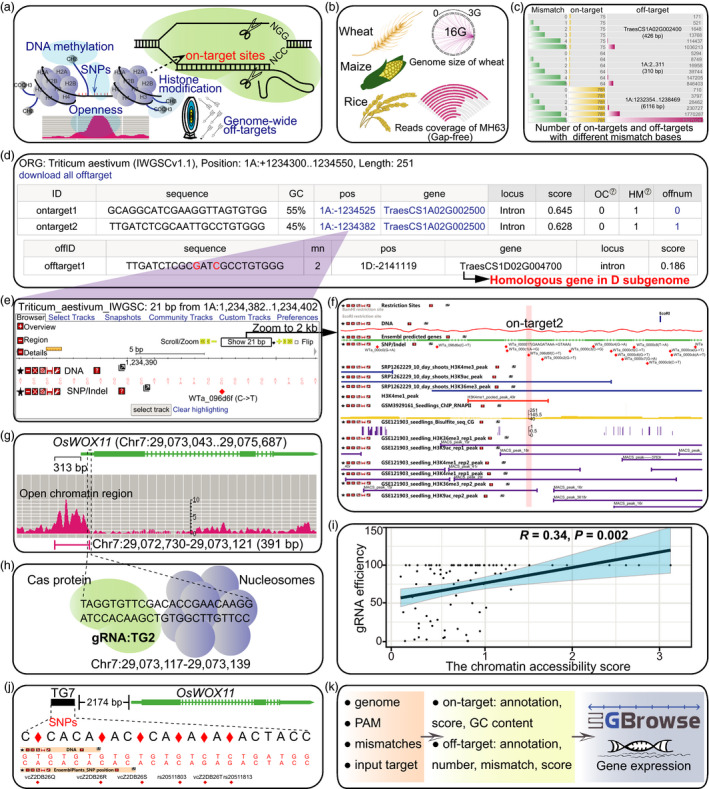
gRNA design using CRISPR‐Cereal tool. (a) Regulome and SNP information are available for on‐targets identified by CRISPR‐Cereal. (b) The crop genomes included in CRISPR‐Cereal. (c) The numbers of genome‐wide off‐targets are increased when allowed mismatches are from zero to five. (d) CRISPR‐Cereal basal result page for gRNA designing. (e) The regulome visualization page using GBrowse. (f) The regulome information in 2 kb region around the on‐target site. (g) The chromatin accessibility on the upstream 350 bp of *OsWOX11*. (h) The chromatin accessibility information on TG2 target region. (i) The correlation between mutant frequency and chromatin accessibility in gRNAs target sites. (j) The SNP information on TG7 target region. (k) The workflow of gRNA design using CRISPR‐Cereal.

Previously, we generated a whole‐genomic pool for scanning gRNA in maize (http://crispr.hzau.edu.cn/CRISPR‐Local/), but this approach is not suitable for wheat which has a 16 Gb genome (Figure 1b), seven times bigger than maize (Appels *et al*., 2018; Jiao *et al*., 2017). It is an obstacle to speedy screen genome‐wide off‐targets in wheat. To solve the problem, CRISPR‐Cereal applies the command‐line tool, FlashFry to perform genome‐wide scan for off‐targets (McKenna and Shendure, 2018). FlashFry uses guide‐to‐genome aggregation model to scan the genome and supports screening for unconstrained number of mismatches for putative off‐targets. Given that DNA cleavage by Cas9 allows three to five mismatches, we made the option of mismatches range from zero to five. CRISPR‐Cereal could identify all the off‐targets with less than four mismatches for each candidate guide in 49 s in wheat, 35 s in maize and 9 s in rice, which outperforms all the other gRNA design tools including E‐CRISP (http://www.e‐crisp.org/E‐CRISP/), CRISP direct (http://crispr.dbcls.jp/) and wheatCRISPR (https://crispr.bioinfo.nrc.ca/WheatCrispr/) in which either only stands two mismatches or cannot search for genome‐wide off‐targets speedily. The output of CRISPR‐Cereal contains information for all off‐targets, which can be downloaded for further comparison. We further compared the off‐targets between CRISPR‐Cereal and Cas‐OFFinder (http://www.rgenome.net/cas‐offinder/), and observed that the off‐targets detected by the above two tools are very similar (details in help page of the website). Significantly, this is the first time to perform genome‐wide off‐target scan in wheat. We randomly submitted three different wheat DNA sequences in FASTA format to search genome‐wide off‐targets with 0–5 mismatches. It shows that the off‐target numbers could reach up to several millions (Figure 1c), implying the importance of whole‐genome scan for off‐targets. The on‐target and off‐target scores are predicted with the widely used on‐target metrics and cutting‐frequency determination (CFD) scoring scheme (Doench *et al*., 2014, 2016). CRISPR‐Cereal provides the information of GC content, position, proximal gene, location of gene structure element (promoter, exon, intron or intergenic), efficiency score of on‐targets and genome‐wide off‐targets (Figure 1d). For the off‐targets in wheat, CRISPR‐Cereal specifies whether they belong to the homologous group in the A, B or D sub‐genomes (Figure 1d), and telling users whether the selected gRNAs would cause unintended editing in the homologous genes from the sub‐genomes.

The importance of regulatory elements such as promoters and distal enhancers in gene expression has been increasingly documented (Yan *et al*., 2019). To ensure more efficient editing of genes or functional elements of interests, we integrated gene expression, chromatin accessibility and epigenetic modifications to assist gRNA design (Figure 1d–f). We collected data sets of assay for transposase‐accessible chromatin using sequencing (ATAC‐seq), DNaseI‐hypersensitivity sequencing (DNaseI‐seq), and formaldehyde‐assisted isolation of regulatory elements by sequencing (FAIRE‐Seq) to locate open chromatin, data from chromatin immunoprecipitation sequencing (ChIP‐seq) to mark histone modifications, and data from whole‐genome bisulphite sequencing (WGBS) to present DNA methylation level. Information of the data sets was listed in http://crispr.hzau.edu.cn/CRISPR‐Cereal/help.php. The data sets were reanalysed and could be easily visualized by Generic Genome Browser (GBrowse) 2.0 (https://github.com/GMOD/GBrowse) after clicking the on‐target position (e.g. 1A:‐1264382) on the elementary result page of CRISPR‐Cereal (Figure 1d,e). Users could extend to a widely region to see the global regulome information around targets (Figure 1f). Besides, to further help users decide which gRNA to use, the chromatin status of the targets has been scored (Figure 1d). The chromatin accessibility information helps to choose and design gRNAs. Recently, Gong and colleagues reported that when gRNA targeted 350 bp upstream of *OsWOX11*, the edit efficiency was high (Gong *et al*., 2020). We found that the reason might be due to the open chromatin feature at that region (Figure 1g). In addition, the gRNA for TG2 that failed to activate transcription is partly located in an un‐open chromatin region (Gong *et al*., 2020) (Figure 1h). To further confirm the relationship between editing efficiency and chromatin accessibility on the gRNAs target sites, we randomly collected the published data for 84 endogenous sites and checked the chromatin accessibility on the corresponding on‐target sites in rice callus (Zhang *et al*., 2012). As expected, gRNAs targeting open chromatin regions result in significantly higher editing efficiency than those against un‐open regions (*R* = 0.34, *P* = 0.002) (Figure 1i).

To expand the application of CRISPR‐Cas tool from reference genome to elite cultivars, CRISPR‐Cereal collected and visualized SNPs information from Ensembl Plants (ftp://ftp.ensemblgenomes.org/pub/plants/release‐48/variation/vcf/) and RiceVarMap v2.0 (http://ricevarmap.ncpgr.cn/) for rice, MaizeSNPDB (https://venyao.xyz/MaizeSNPDB/) for maize and 487 wheat genotypes (Pont *et al*., 2019). Remarkably, we found that 39.59% guide sequences in rice genome possess SNPs, which would cause mismatches if guide sequence would be decided only by reference genome. One example is that the gRNA for TG7 designed based on the Nipponbare genome has 6 SNPs failed to mediate transcription activation of *OsWOX11* (Gong *et al*., 2020) (Figure 1j), although the location and chromatin openness may also play roles in this process.

In summary, CRISPR‐Cereal integrates regulome information and considers SNPs existed in the candidate gRNAs to promote precise and high‐efficient gene editing for wheat, maize and rice. The workflow of CRISPR‐Cereal is shown in Figure 1k, it is freely available at http://crispr.hzau.edu.cn/CRISPR‐Cereal/.

## Conflict of Interest

The authors declare no conflict of interest.

## Author contributions

C.H. and H.L. collected the data and built the gRNA design platform and GBrowse visualization platform. C.H. drafted the manuscript with input from L.‐L.C. and W.Y. D.C. analysed the histone modifications data of wheat and rice. W.‐Z.X. analysed the genomic variation data of rice. M.W., Y.L. and X.G. contribute to generate the web‐page. L.‐L.C. and W.Y. conceived of the study and wrote the manuscript. All authors read and approved the final manuscript.

## Funding

This work was supported by the National Transgenic Science and Technology Program (2019ZX08010‐003), the National Natural Science Foundation of China (31871269), Hubei Provincial Natural Science Foundation of China (2019CFA014) and the Fundamental Research Funds for the Central Universities (2662020ZKPY017).
